# RhTFAM treatment stimulates mitochondrial oxidative metabolism and improves memory in aged mice

**DOI:** 10.18632/aging.100488

**Published:** 2012-09-30

**Authors:** Ravindar R. Thomas, Shaharyar M. Khan, Rafal M. Smigrodzki, Isaac G. Onyango, Jameel Dennis, Omer M. Khan, Francisco R. Portell, James P. Bennett

**Affiliations:** ^1^ Parkinson's Disease Center, Virginia Commonwealth University, Richmond, VA 23298, USA; ^2^ Gencia Corporation, Charlottesville, VA 22903, USA; ^3^ Department of Neurology, Virginia Commonwealth University, Richmond, VA 23298, USA

**Keywords:** aging, mitochondrial DNA, mitobiogenesis, recombinant human TFAM

## Abstract

Mitochondrial function declines with age in postmitotic tissues such as brain, heart and skeletal muscle. Despite weekly exercise, aged mice showed substantial losses of mtDNA gene copy numbers and reductions in mtDNA gene transcription and mitobiogenesis signaling in brain and heart. We treated these mice with weekly intravenous injections of recombinant human mitochondrial transcription factor A (rhTFAM). RhTFAM treatment for one month increased mitochondrial respiration in brain, heart and muscle, POLMRT expression and mtDNA gene transcription in brain, and PGC-1 alpha mitobiogenesis signaling in heart. RhTFAM treatment reduced oxidative stress damage to brain proteins, improved memory in Morris water maze performance and increased brain protein levels of BDNF and synapsin. Microarray analysis showed co-expression of multiple Gene Ontology families in rhTFAM-treated aged brains compared to young brains. RhTFAM treatment reverses age-related memory impairments associated with loss of mitochondrial energy production in brain, increases levels of memory-related brain proteins and improves mitochondrial respiration in brain and peripheral tissues.

## INTRODUCTION

Diseases of aging represent substantial socioeconomic burdens for modern societies that are increasingly composed of aged individuals. Aging in postmitotic tissues such as brain, heart and skeletal muscle increases risk of neurodegenerative diseases, cardiomyopathy with heart failure and sarcopenia, respectively. Overcoming these impairments would improve quality of life for aged individuals and markedly lessen burdens on caregivers and societies.

The mitochondrial theory of aging (MTA) is particularly relevant to understanding age-related diseases in these post-mitotic tissues. In a broad formulation, this theory posits that aging leads to increasing deficits in bioenergetic capacities of mitochondria such that cellular energy requirements are not met. These bioenergetic deficits not only decrease energy production capacity but also increase damage from oxygen free radical production. Such bioenergetic deficits could arise from impaired signaling for maintaining intracellular mitochondrial mass (mitochondrial biogenesis; “mitobiogenesis”), abnormal assembly of mitochondrial energy production systems, increased damage from reactive oxygen/nitrogen species to mitochondrial components, altered fission/fusion control, increased mitochondrial destruction (mitochondrial autophagy; “mitophagy”), or some combination of processes.

Earlier formulations of the MTA emphasized mitochondrial production of ROS as the primary catalyst for aging [1, 2]. That concept has become increasingly controversial [3-7], and other explanations for age-related mitochondrial bioenergetic losses are being sought [8, 9]. One emerging theme is that ROS serve an important signaling role [10-12], not so much a detrimental role, and the mammalian target of rapamycin (mTOR) kinase pathway contributes to aging, possibly through producing cellular hyperfunction [13, 14].

Mtiochondrially targeted treatment has shown some successes in improving lifespans of various organisms [15, 16]. This finding is consistent with observations of mitochondrial deficiencies in aging Ames mice [17] and involvement of oxidative stress [18, 19] and mitochondrial bioenergetics [20] in cardiac failure.

To treat mitochondrial deficiencies, we have developed recombinant human mitochondrial transcription factor A (rhTFAM) [21-24]. TFAM is an essential component of the mitochondrial DNA replication and expression machinery and contains two high mobility group (HMG) domains that bind to mtDNA. RhTFAM includes an N-terminal protein transduction domain to allow rapid translocation across cell membranes, followed by an SOD2 mitochondrial localization signal to stimulate uptake through the TOM-TIM mitochondrial translocases [21]. RhTFAM enters the mitochondrial compartment of cells rapidly and can also transport mtDNA cargo into mitochondria [21, 25]. RhTFAM stimulates mitochondrial biogenesis of human cells modeling sporadic Parkinson's disease [23] or containing high abundance mtDNA mutations of Leber's hereditary optic neuropathy (LHON) or Leigh syndrome [25]. RhTFAM treatment of cells exposed to parkinsonian neurotoxins restores ATP deficiencies and reduces oxidative stress [24]. Systemic treatment of young adult mice with rhTFAM stimulates mitochondrial biogenesis, increases respiration in brain, heart and muscle, increases brain mitochondrial ATPsynthesis and reduces oxidative stress damage to proteins [21, 24].

These desirable properties of rhTFAM suggest that it might improve bioenergetic deficiencies produced as a consequence of aging. To test that possibility, we treated aged mice with rhTFAM in a manner similar to our prior study of treating young adult mice [21, 24]. We observed stimulation of mitochondrial biogenesis and mtDNA gene expression in the absence of any apparent systemic toxicity. Increases in mitochondrial oxidative metabolism were mirrored by improvements in Morris water maze performance in aged mice, including platform acquisition (learning) and platform location recall (memory), and increases in brain protein levels of BDNF and synapsin. These findings support beneficial use of rhTFAM in human aging and development for experimental use of rhTFAM in humans.

## RESULTS

### Effects of aging and rhTFAM treatment on mtDNA gene copy numbers

We used multiplex qPCR for four mouse mtDNA genes (16S rRNA, CO1, CO3, CytB) normalized to the geometric means of three abundantly expressed housekeeping genes (GAPDH, beta-actin, 18S rRNA) in each sample to improve precision of our assays. By comparing mtDNA gene levels between comparable tissues of buffer CTL-treated 5 month-old young adult (n=6) and 21 month-old aged mice (n=9), we obtained estimates of the effects of aging in these tissues from male mice who had undergone vigorous, once weekly rotarod exercise testing over 4 weeks.

**Figure 1:**
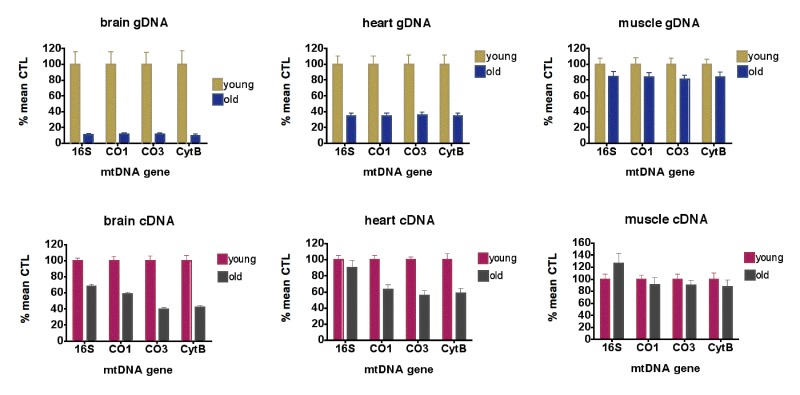
Aging causes loss of mtDNA copy numbers and mtDNA transcription (**top row**) qPCR analyses of mtDNA gene levels in gDNA from young (5 month, n=6) and old (21 month, n=9) brains (left), hearts (middle) and skeletal muscle (right), expressed as % mean young mouse levels. For brains and hearts, all differences between young and old animals are significant at p<0.0001; for muscle, differences between young and old CO3 levels were significant at p=0.036. No other genes had significant differences between young and old animals (all by unpaired t-test). (**bottom row**) qPCR analyses of mtRNA gene levels as cDNA?s in young (5 month, n=6) and old (21 month, n=9) brains (left), hearts (middle) and skeletal muscle (right), expressed as % mean young mouse levels. For brains, all differences between young and old animals are significant at p<0.0001; for heart, all differences between young and old are significant at p=0.001 or less; for muscle, differences between young and old are not significant (all by unpaired t-test).

As shown in Figure 1 (top row), aging of the mice from 5 months to 21 months resulted in losses of mtDNA gene levels of 88.6-90.3% in brain, 65.4-66.0% in heart and 16.7-19.4% in skeletal muscle. RhTFAM treatment did not significantly change mtDNA gene levels in heart and muscle, and resulted in 28.0-32.8% losses in brain relative to buffer control treatment in exercised aged mice (not shown).

### Effects of aging and rhTFAM treatment on mtDNA gene expression

qPCR using normalization to geometric means of the same housekeeping genes on cDNA samples from 5 month-old and 21 month-old mice showed that levels of 16S rRNA were elevated 10-fold or greater compared to mtDNA protein coding genes (CO1, CO3, CytB) in all samples (not shown). For mtDNA coding genes, aging reduced expression 41.0-60.7% in brain, 37.1-44% in heart and 9.6-12.5% in skeletal muscle (Figure 1, bottom row). In aged mice, rhTFAM treatment increased mtDNA gene expression 42-97% in brain when compared to buffer CTL, suggesting rescue of mtRNA lost due to aging (Figure 3, left).

### Effects of aging and rhTFAM treatment on mitobiogenesis signaling

Mitobiogenesis signaling is a complex system of interacting transcription factors and co-activators that regulate expression of many mitochondrial genes involved in energy metabolism and respiration [26]. The major known upstream regulator is peroxisome proliferator-activated receptor gamma, coactivator 1-alpha (PGC-1α) that is believed to control the expression of many energy metabolism genes though regulation of nuclear respiratory factors (NRF) 1 and 2 [26]. In addition to expression of PGC-1α, we assayed expression of TFAM, mitochondrial transcription factor B2 (TFMB2) and nuclear respiratory factor 2 (NRF2), important downstream targets of PGC-1α.

**Figure 2:**
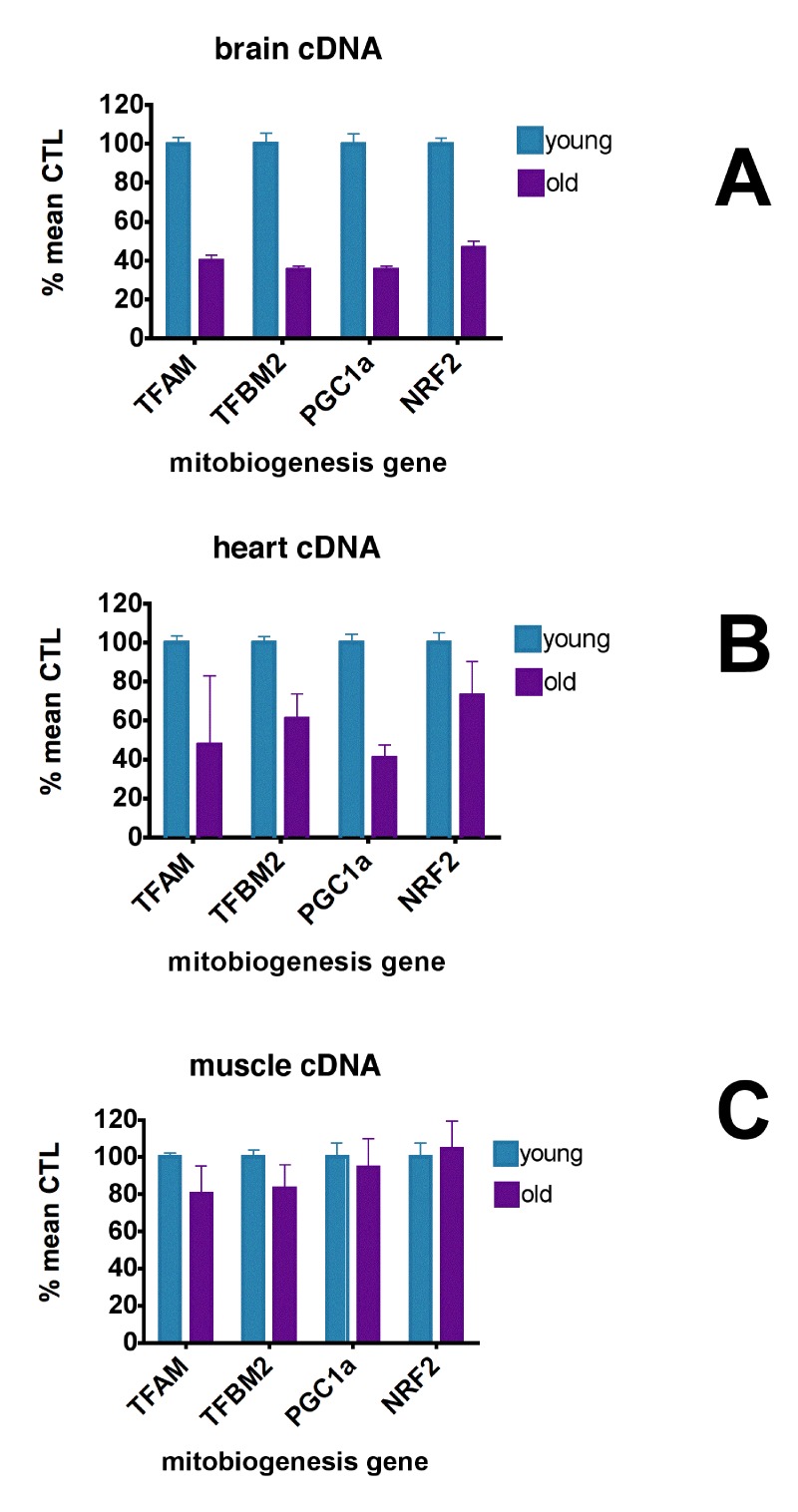
Aging causes loss of mitochondrial biogenesis signaling qPCR analyses of mitobiogenesis gene expression levels in cDNA's from young (5 month, n=6) and old (21 month, n=9) brains (**A**), hearts (**B**) and skeletal muscle (**C**), expressed as % mean young mouse levels. In brain, expression of all genes in young brains were different from that in old brains at p<0.0001 (unpaired t-test). In heart, expression of TFBM2 (p=0.0325) and PGC-1α (p<0.0001) were significantly different between young and old animals (unpaired t-test). Expression of TFAM and NRF2 were not significantly different. In muscle, expression of TFAM (p=0.012) and TFBM2 (p=0.036) in young mice were significantly different from old mice; expression of PGC1α and NRF2 were not significantly different between young and old mice (unpaired t-test).

**Figure 3:**
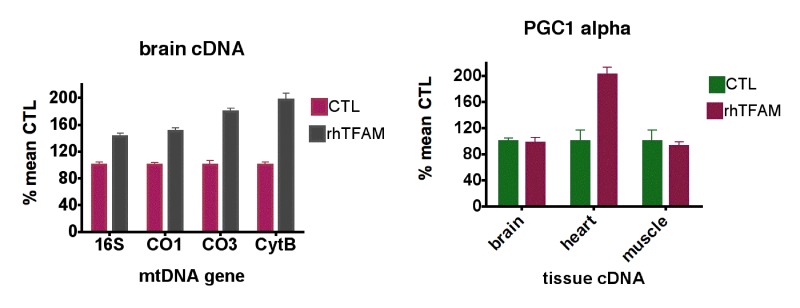
RhTFAM treatment increases transcription of mtDNA and mitobiogenesis genes (**left**) qPCR analyses of brain mtRNA gene levels as cDNA's in old (21 month) mice treated with rhTFAM or buffer CTL (n=9 each group), expressed as % mean CTL levels. For brains, all differences between young and old animals are significant at p<0.0001 (paired t-test). (**right**) Effects of rhTFAM on expression of PGC-1α in brain, heart and skeletal muscle of old (21 month) mice, expressed as % of mean CTL treatment values (n=9 each group). In heart, rhTFAM significantly elevated PGC-1± expression (p=0.0022 by paired t-test).

Figure 2 shows that levels of expression of these mitobiogenesis genes were reduced 55.6-64.8% in brain and 27.1-59.3% in heart compared to young CTL mice but were not reduced in skeletal muscle. Interestingly, rhTFAM treatment did not elevate these transcription factor mRNA's in brain but did elevate heart PGC-1α expression by 102% (Figure 3, right) and NRF2 expression by 30% (not shown). According to our observations, the age-related losses of mtDNA copy numbers and gene expression levels in brain and heart may derive from reductions in mitobiogenesis signaling in these tissues.

### Effects of aging and rhTFAM treatment on expression of brain DNA polymerase gamma (POL-γ) and mitochondrial DNA-directed RNA polymerase (POLRMT)

MtDNA is replicated by DNA polymerase gamma (POL-γ), and mitochondrial DNA-directed RNA polymerase (POLRMT) mediates both synthesis of RNA primers necessary for mtDNA replication and mtDNA gene transcription. Given the substantial age-related loss in brain of mtDNA gene copy numbers (~90%) and mtDNA gene expression (~40-60%), we then sought to determine if aging reduced expression of POL-γ or POLRMT.

**Figure 4:**
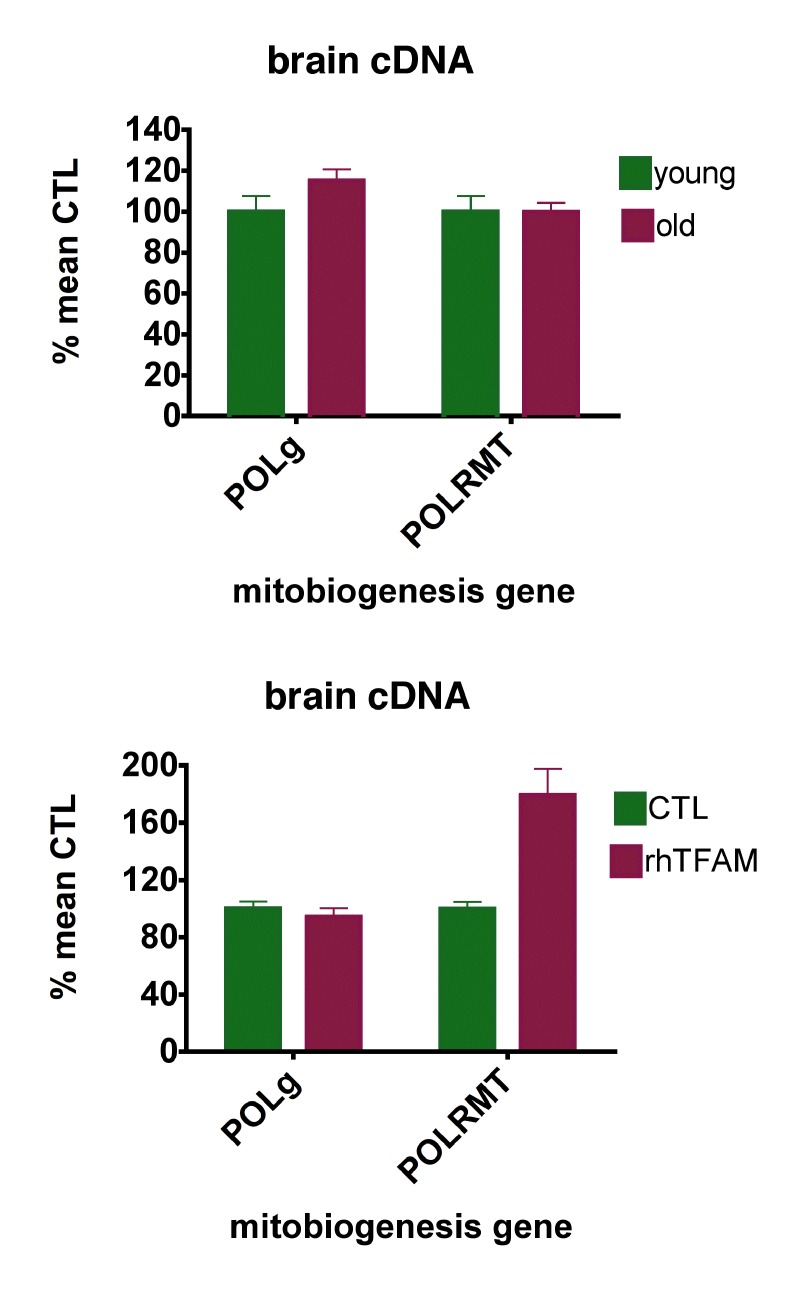
RhTFAM treatment increases expression of brain mitochondrial RNA polymerase (POLRMT) (**top**) expression of POL-g and POLRMT in brains of young (5 month, n=6) and old (21 month, n=9) mice. There were no significant differences. (**bottom**) effects of rhTFAM on expression of POL-g and POLRMT in brains of old (21 month) mice (n=9 each group). RhTFAM treatment increased expression of POLRMT (p=0.0045 by paired t-test).

Figure 4 (top) shows that aging did not meaningfully alter brain expression levels of POL-γ or POLRMT. RhTFAM treatment of aged mice did not change brain POL-γ expression but increased expression of brain POLRMT by 79%, commensurate with the increases in mtRNA (Figure 4 bottom).

### Effects of aging and rhTFAM treatment on expression of brain SirT3

**Figure 5:**
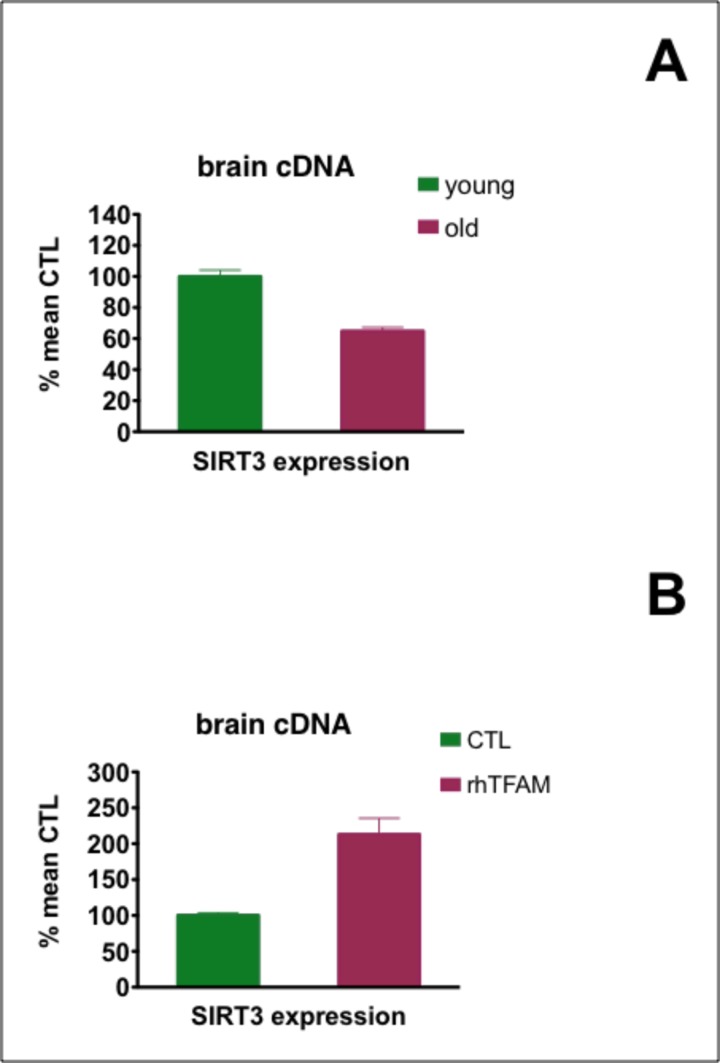
Effects of aging and rhTFAM treatment of aged mice on expression of brain Sirt3 In (**A**), expression of Sirt3 in young (n=6) compared to old (n=90 brains was significant at p<0.0001 by unpaired t-test. In (**B**), rhTFAM treatment (n=9) significantly increased Sirt3 expression compared to CTL treatment (n=9) in aged mice at p=0.0012 by unpaired t-test.

Mammalian tissues possess seven different sirtuins that are NAD+ dependent de-acetylating and ADP ribosyl transferase enzymes [27]. Sirtuins 3,4 and 5 have mainly mitochondrial localizations, with sirtuin 3 (Sirt3) believed to function as the major mitochondrial de-acetylase [28, 29]. We found that Sirt3 expressionwas significantly reduced ~40% in old compared to young mouse brains. RhTFAM treatment increased aged brain expression of Sirt3 over two-fold to 80% of the levels found in young mouse brains (Figure 5).

### Effects of Treating Aged Mice with rhTFAM on Brain Gene Expression Changes in Gene Ontology (GO) Families Compared to Aging Brain

We created pooled RNA samples composed of equal amounts of total RNA from the brains of 6 CTL young (5 month old) mice, 9 CTL aged (21 month old mice) and 9 rhTFAM-treated aged mice. With Illumina microarrays, we analyzed in independent triplicates these pooled RNA samples for gene expression and used GeneSpring GX to process the data. We searched the “entity list” for genes expressed with >3-fold changes in the rhTFAM treatment of aged mice compared to the Gene Ontology families identified (q<0.1) in the young compared to aged CTL mouse brains. As shown in Table 1, we found highly significant overlap among genes with >3-fold differential expression in aged mice treated with rhTFAM and 19 GO families differentially expressed in aged compared to young adult brains. 15 of these GO families relate to developmental and morphogenesis gene families; 4 relate to DNA binding and transcription factor activities.

**Table 1:** Matched Entity Lists for gene ontology (GO) co-expression in 5 month-old/21 month-old brains compared to 21 month-old rhTFAM/CTL-treated brains

Entity List	p value
developmental process	4.64E-11
multicellular organismal development	6.47E-11
anatomical structure development	5.54E-11
DNA binding	7.93E-11
extracellular region	5.50E-11
system development	8.19E-11
organ development	1.02E-10
sequence-specific DNA binding transcription factor activity	1.72E-09
nucleic acid binding transcription factor activity	1.72E-09
sequence-specific DNA binding	3.42E-06
pattern specification process	5.90E-04
regionalization	0.0015124
embryonic organ development	0.001255763
skeletal system development	8.41E-04
anterior/posterior pattern formation	5.37E-04
embryonic organ morphogenesis	0.003916774
embryonic skeletal system development	0.001636222
skeletal system morphogenesis	0.001260297
embryonic skeletal system morphogenesis	0.00653228

### RhTFAM increases brain levels of mitochondrial electron transport chain subunits, Complex I and IV activities, ATP and reduces oxidative stress

**Figure 6:**
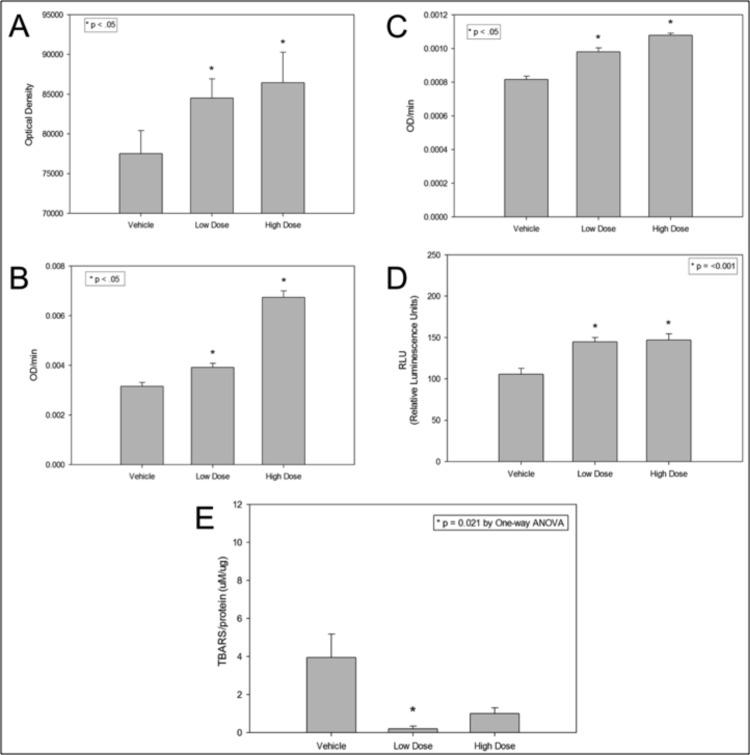
RhTFAM treatment improves brain mitochondrial functions and lowers oxidative stress damage to brain proteins and lipids Effects of rhTFAM on various mitochondrial parameters in aged mice. Brain mitochondrial pellets from rhTFAM (low and high dose) and vehicle treated 21-22 month old mice were assayed for levels of various OXPHOS subunits (**A**), Complex I and IV activities (**B** and **C**, respectively), total ATP (**D**) and TBARS (**E**) as a measure of oxidative stress. RhTFAM treatment significantly increased all mitochondrial measures except for oxidative stress, where the low dose produced a significant decline in levels of TBARS. Unless otherwise noted, * p<.05 by paired t-test.

Brains of aged mice from the MWM study were lysed and P2 mitochondria-enriched fractions prepared. We determined the effects of rhTFAM on individual brain electron transport chain subunits. Four micrograms of brain P2 fractions from each brain were run on SDS-PAGE and transferred for blotting. Using the MitoProfile® Total OXPHOS rodent antibody cocktail (ab110413, Abcam), optical density units of the five bands were totaled. RhTFAM produced a 60% increase in the levels of the various subunits when compared to vehicle treated aged mice (Figure 6A). The aged mouse brain P2 mitochondria enriched fractions were assayed for Complex I and IV activities as well as total ATP. RhTFAM treated brain mitochondria had significantly higher enzyme activities as well as total ATP (Figure 6B,C,D).

Brain lysates were also studied for levels of TBARS (Thiobarbituric Acid Reactive Substances) a measure of ROS driven lipid peroxidation using the OxiSelect TBARS assay kit (Cell Biolabs). The Low Dose rhTFAM group had significantly less brain lipid peroxidation byproducts and the High Dose rhTFAM group trended towards the same as compared to the Vehicle group (Figure 6E).

### Mitochondrial respiration of rhTFAM treated aged mice exercised on rotarod

In this study carried out in aged 20-21 month-old male mice, we used a different motor assessment protocol that emphasized time on a slowly accelerating rotarod instead of repeated, constant velocity rotatorod testing to near-exhaustion used in our earlier study of young mice [21, 24]. One week after the last rhTFAM injection and rotarod testing of aged mice, we sacrificed them in pairs (CTL and rhTFAM, n=9 treatment pairs), removed brain, heart and quadriceps muscle, prepared P2 mitochondrial pellets and assayed respiration capacity in these mitochondrial preparations by sequentially providing substrates for specific respiratory complexes (CI, CII, CIV). We found that rhTFAM treatment brought about significant increases in mitochondrial respiration in brain (CI, CIV), heart (CI, CIV) and skeletal muscle (CI) (Figure 8).

**Figure 7:**
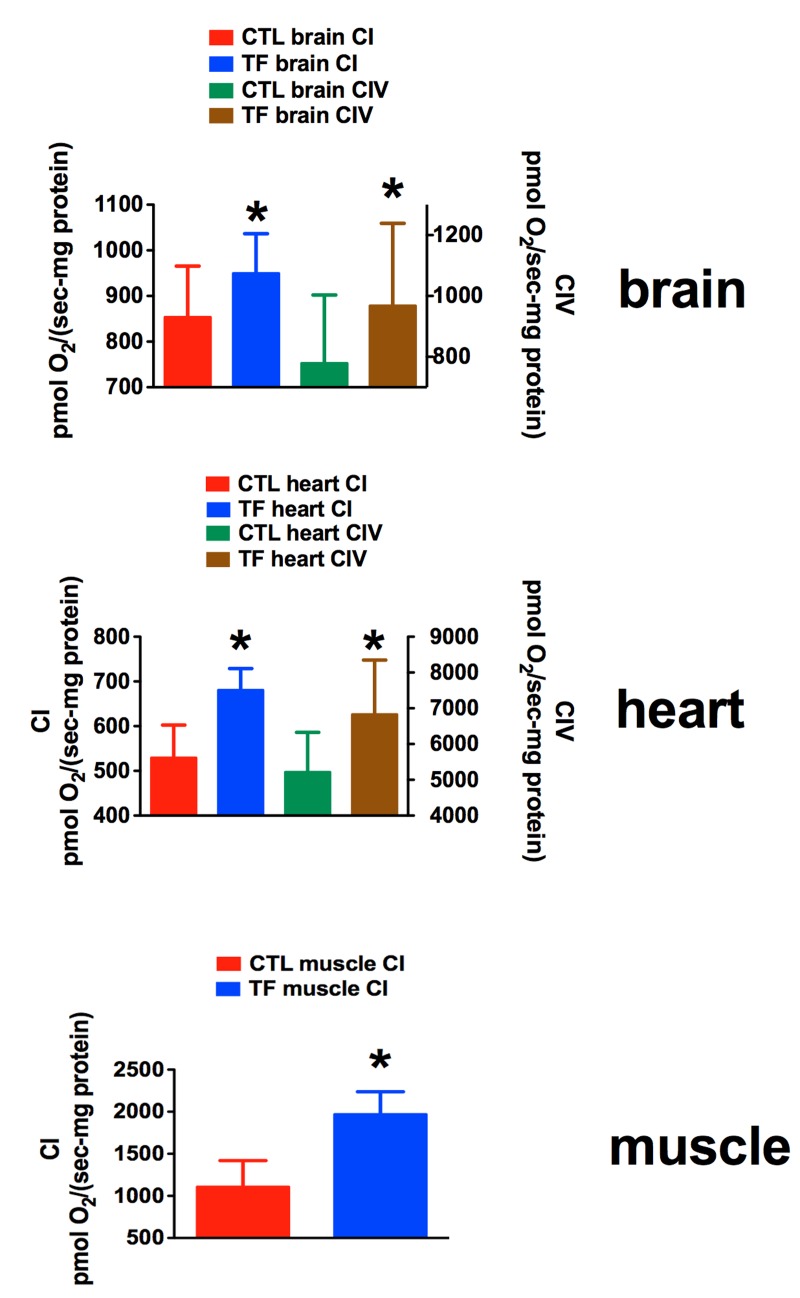
RhTFAM treatment improves State 3 mitochondrial respiration in brain, heart and skeletal muscle Effects of rhTFAM treatment on state 3 (+ADP) respiration in mitochondrial preparations from brain, heart and muscle in 21 month old mice treated weekly with rhTFAM or buffer CTL, following addition of substrates for Complex I (CI) or Complex IV (CIV). * p<0.05 by paired t-test.

**Figure 8:**
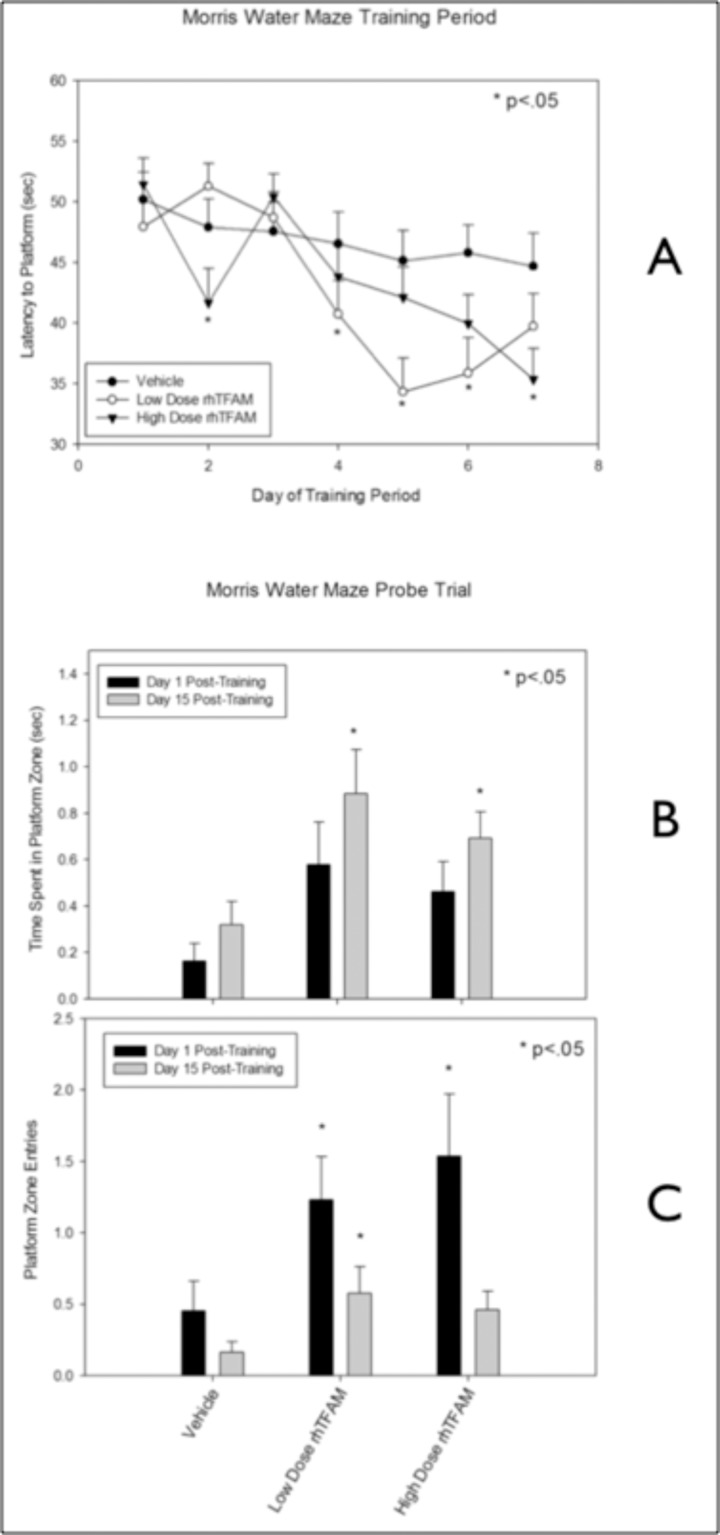
RhTFAM treatment improves learning and memory in aged mice (**A**) Effects of rhTFAM and vehicle on latency to platform on during the seven days of Morris Water Maze training in aged (21-22 month old) mice. RhTFAM treatment significantly improved latency to platform (p<.05 by paired t-test). One and fifteen days after training, the platform was removed and mice were tested to determine recall of the position of the platform. RhTFAM treated aged mice spent significantly more time (**B**) in the platform zone as well as entering the zone more often (**C**) than did vehicle treated aged mice, * p<.05 by paired t-test.

### Effects of rhTFAM treatment of aged mice on Morris Water Maze

20 month-old mice were divided into three groups, vehicle, low and high dose rhTFAM and treated weekly via intravenous tail vein injections for a total of 4 doses. During Week 5, Morris water maze (MWM) training was initiated. The platform was submerged below the surface of the water, and the mouse was placed in the water and given 60 seconds to locate the platform. Once on the platform, mice were allowed to remain for 15 seconds before being returned to their cage. Each mouse underwent four successive trials a day, with 10-minute intervals, for 7 days. Latency (seconds) was averaged by each run (4 runs per timepoint), and then by treatment group. Both rhTFAM treated groups acquired the platform faster and earlier than did the vehicle treated aged mice (Figure 8A).

To measure the strength of spatial memory retention, on the 8th day, a ‘probe test’ was conducted. Mice were placed in the water to swim freely for 60 seconds without the platform and the time spent in the quadrant that had contained the platform, and the number of crosses over the previous position of the platform, was recorded. The probe trial was repeated 15 days later. Both rhTFAM treated groups spent significantly more time in the platform zone (Figure 8B) as well as entered the zone more often at the 8th day (Day 1 post-training) as well as the 15th day post-training (Figure 8C).

### rhTFAM treatment of aged mice dose-dependently increases brain protein levels of BDNF and synapsin

Synaptic plasticity, learning and memory are associated with brain-derived neurotrophic factor (BDNF) signaling that occurs through its tyrosine kinase receptor TrkB. BDNF expression is driven by CREB binding to its promoter IV [30], which is notable given that the canonical PGC-1 alpha promoter also has a CREB site, suggesting that expressions of both BDNF and PGC-1-alpha could be driven by CREB activation. Expressions of both PSD-95 [31] and synapsin [32-34], both abundant synaptic proteins, are driven by BDNF signaling. Hippocampal BDNF signaling through its TrkB receptor mediates exercise-induced improvements in memory [33].

**Figure 9:**
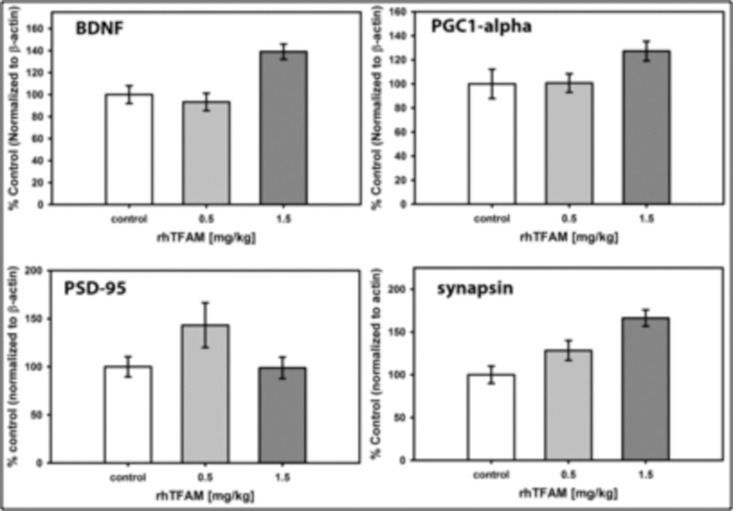
RhTFAM treatment increases brain levels of BDNF and synapsin Brain levels of proteins assayed by Western blot after treatment of 21 month old mice with rhTFAM once/week for four weeks. Shown are sizes of single rhTFAM doses. Protein levels are normalized to beta-actin and are expressed as % mean CTL. Statistical differences: BDNF (p=0.028 (control v 0.5mg/kg), p=0.029 (control v. 1.5mg/kg), p<0.001 (0.5mg/kg v 1.5mg/kg); PGC-1a (NS); PSD-95 (NS); synapsin (p<0.001 (control v. 1.5mg/kg), p=0.005 (0.5mg/kg v. 1.5mg/kg))

To determine whether the rhTFAM-induced increases in memory function were associated with changes in BDNF, PSD-95 or synapsin expression, we assayed levels of these proteins and PGC-1-alpha in brains of old mice treated weekly for four weeks with rhTFAM or buffer CTL. As shown in Figure 9, BDNF and synapsin proteins increased dose-dependently. These results support the interesting possibility that rhTFAM treatment both increases mitobiogenesis in brain (through increas-ing PGC-1α) and stimulates memory improvement by increasing expression of BDNF (possibly through CREB) that then increases expression of the important synaptic protein synapsin involved in memory formation.

### Young mice treated with rhTFAM have improved learning

**Figure 10:**
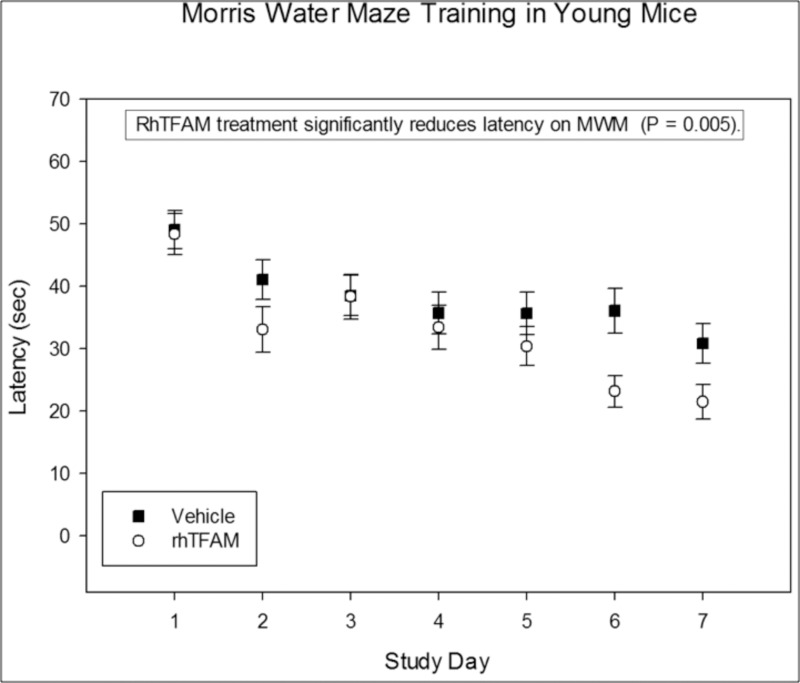
RhTFAM treatment improves learning in young mice. Effects of rhTFAM and vehicle on latency to platform during the seven days of Morris Water Maze training in young (2-3 month old) mice. RhTFAM treatment significantly improved latency to platform (p<.05 by paired t-test).

In a separate study, twenty 8 to 10 week old male C57BL/6J mice were assigned to two groups, vehicle control (n=10) and rhTFAM (n=10). As in our aging studies, mice were dosed IV weekly for 4 weeks with rhTFAM or vehicle. At the completion of dosing Morris water maze training was begun for one week using the same protocol as for the aged mouse study. RhTFAM treatment significantly improved platform acquisition time in young mice suggesting increasing mitochondrial bioenergetics impacts cognition regardless of age (Figure 10). Additionally, vehicle treated young mice acquired the platform in ~31±3 sec at the completion of the 7 day training period. Aged mice treated with rhTFAM acquired the platform in ~35±3 sec at the same point in training; latency was not significantly different between control young mice and rhTFAM aged mice. Though from two separate studies conducted in the same way by the same personnel, the comparison suggests that rhTFAM treatment may rejuvenate cognition in the aged and improve cognition in the young.

## DISCUSSION

By using qPCR normalized to geometric means of three major housekeeping genes, we have shown that aging in exercised mice results in substantial losses of mtDNA gene copy numbers, mtDNA gene expression and mitobiogenesis signaling in brain and heart with minimal changes in skeletal muscle. We observed the greatest age-related losses of all categories of genes related to mitochondrial function in brain. If our findings reflect the consequences of the mitochondrial theory of aging, then brain is the most vulnerable post-mitotic tissue of those examined. Age-related losses of mitochondrial gene regulation in brain are profound, and compensatory mechanisms we uncovered are minimal. On a relative basis, the expression of mtDNA genes is increased relative to the loss of mtDNA gene copy numbers, but this is not sufficient to compensate.

A study in the short-lifespan, annual fish *N. furzeri* showed that aging resulted in ~50% losses of mtDNA copy numbers in brain and ~40% losses in muscle [35]. Muscle from aged fish had reduced expression of PGC-1α (expression in brain not reported) and ~70% losses of mitochondrial state 3 respiration driven by CI or CII substrates [35]. In rat brain, aging from 3 months to 36 months of age produced ~40% losses of State 3 mitochondrial respiration at CI and CII and ATP synthesis rates [36]. Our analysis of cerebral cortex was fortuitous as Dinardo et al. had found no deficit in either levels of mtDNA or TFAM in aged (26 versus 5 month) rat cerebellum, liver or kidney. The differences between brain regions may reflect the inherent higher levels of cerebral versus cerebellar mitochondrial transcription rates, oxidative and nitrosative stress, permeability transition and complex I activities, particularly in aging human brain [37-39]. Aging in different vertebrate organisms appears to exact a similar price of decreased cerebral mitochondrial function that appears to arise from reduced mitobiogenesis signaling.

It is unclear why brain should be the tissue most vulnerable to these age related losses, and it is not at all obvious why mitobiogenesis is so impaired during aging. Genes downstream of PGC-1α could be downregulated in response to the reduced expression of PGC-1a we found in brain and heart, but this does not provide understanding of why expression of PGC-1α is so reduced, apparently as a primary bioenergetic deficit in aged brain. Reduced PGC-1α expression could arise from microRNA's (www.microrna.org lists 11 miRNA's that could interact with mouse PGC-1α). Of the 11 miRNAs that may interact with PGC-1α, mir-451 is predicted to interact with mtDNA in the mitochondrial matrix and has been shown to be up-regulated in individuals who fail to generate increased muscle mass in response to resistance training [40-42]. PGC-1α activity can also be regulated by gene methylation or promoter inhibition. A recently described example of the latter is parkin-interacting substance (PARIS), a zinc-finger protein that binds to the insulin-responsive sequence of PGC-1α and acts as a transcriptional repressor, at least in human cells [43]. Neither PARIS status in aged mouse brain nor PGC-1α gene methylation levels or promoter chromatin binding changes have been investigated so far.

Our results with rhTFAM show that repeated treatment can increase mitochondrial respiration in brain, heart and muscle even in the backdrop of exercise. These increases in brain mitochondrial respiration relate to increased mtDNA gene expression (without detected changes in mitobiogenesis signaling genes), and in heart to increases in mitobiogenesis signaling genes (without detected changes in mtDNA gene expression assayed at the completion of dosing). Thus, there may be differing mechanisms underlying rhTFAM effects in these different tissues, with the same net result of increased mitochondrial respiration and energy production capacity.

Levels of TFAM protein within mitochondria appear to be regulated by *Lon* protease. RNAi knockdown of *Lon* increases mitochondrial TFAM levels, resulting in increased mtDNA copy number [44]. We do not know if levels of mitochondrial *Lon* or TFAM were altered in our aged mouse cerebrum. Because we found brain TFAM gene expression was reduced ~60% by aging, invoking post-translational regulation of TFAM, such as by *Lon* degradation, does not appear to be necessary to explain our findings.

Human TFAM expressed in mouse hearts with genetic deletion of their endogenous cardiac TFAM mature normally, are healthy and do not express a cardiomyopathy phenotype. Hearts from these cardiac TFAM-deleted mice “rescued” by human TFAM expression contain near normal levels of mtDNA gene transcripts, in spite of the marked reduction of *in vitro* mouse mtDNA transcription initiation by human TFAM. These findings suggest that human TFAM expression in mice can promote normal levels of mtDNA gene transcripts through a mechanism likely different than induction of mtDNA transcription alone. As discussed by Freyer, et al, [45] this mechanism could include post-transcriptional mtRNA stabilization.

It is conceivable that a similar mechanism might be operative in our mice given once weekly injections of rhTFAM that targets the mitochondrial matrix and would be processed to mature human TFAM. In particular, we found that rhTFAM treatment produced an increase in mtDNA transcripts in aged mouse brains that at baseline showed ~60% reductions (relative to young adults) in expression of these critical respiratory complex genes. Alternatively, this increase in mtDNA-encoded gene expression could be the result of increased POLRMT (mitochondrial RNA polymerase) we detected in rhTFAM treated aged mice. We observed this increase in mtDNA gene transcripts in aging brain in the setting of marked loss (>90%) of brain mtDNA gene copy numbers. This degree of loss was comparable to that observed in mouse hearts where TFAM was deleted and not replaced (see Fig 4B, [45]). In young adult mice rhTFAM treatment can increase mtDNA gene copy numbers [24]. It would be interesting to study whether the reduction in mtDNA gene copy could be reversed in aged mice given alterations to the rhTFAM dosing regimen, or whether this phenomenon reflects a fundamental change in rhTFAM effects on mtDNA replication in aged compared to young adult mice.

Recent studies have shown that mitochondrial energy producing, oxidative phosphorylation proteins are extensively acetylated on lysine residues [46]. De-acetylation by Sirt3 appears to increase respiration and decrease reactive oxygen species production [29]. Sirt3 expression is regulated by PGC-1α, which may explain our finding of decreased Sirt3 expression in aged brains [47, 48]. However, the rhTFAM-induced increase in aged brain Sirt3 expression occurred without any detectable changes in expression of PGC-1α, so it is not clear how Sirt3 expression was increased. Our observed increases in brain mitochondrial respiration after rhTFAM treatment may have arisen in part from deacetylation of respiratory proteins mediated by increased Sirt3.

The literature is replete with studies showing an association between deficits in mitochondrial oxidative metabolism and reductions in cognition. Because of the dearth of molecular entities that can increase mitochondrial OXPHOS in the brain, few studies have shown that increased mitochondrial oxidative metabolism can yield increases in cognitive function. Utilizing a battery of cognitive tests and measures of hippocampal synaptic transmission, Hayashi et al. showed that over-expressing human TFAM in microglia of mice reversed cognitive deficits produced as a consequence of age (2 versus 24 months of age) [49]. That both this study and Hayashi et al. utilized human TFAM in mice and that human TFAM is less potent in activating mouse mtDNA transcription suggest greater effects maybe seen in humans with rhTFAM. Regardless, our studies indicate that rhTFAM can produce significant improvements in cognitive function without the need for genetic modification and over a relatively short period of time. Furthermore, the magnitude of the rhTFAM observed effect compares favorably to the effects of approved cognition enhancing drugs such as donepezil, galantamine and modafinil, or the cognitive benefits of exercise or caloric restriction reported in the literature [50-54]. Our observed increases after rhTFAM treatment in brain BDNF and synapsin, proteins important for memory function, may participate in the improved water maze memory demonstrated by the aged mice.

In summary, we find that aging in mice produces marked losses in brain (and to a lesser degree in heart) of mitochondrial DNA gene copy numbers. Mitobiogenesis signaling is substantially reduced in aging in brain and heart. Treatment with the novel, mitochondrially targeted protein, rhTFAM, increases mitochondrial respiration in brain, heart and skeletal muscle of aged mice. In aged brain, rhTFAM treatment increased POLRMT expression, which may explain the increases we observed in mtDNA gene expression relative to the profound loss of mtDNA copy numbers produced as a consequence of age. RhTFAM treatment also increased respiratory proteins and greatly reduced levels of ROS damage in aged brain. These biochemical changes were correlated with significant improvements in cognition and increases in brain levels of BDNF and synapsin.

It is intriguing to consider the possibility of combined treatment with rhTFAM and rapamycin, an mTOR inhibitor. If aging deficits arise from a combination of reduced mitobiogenesis signaling with respiratory impairments, and overactivation of mTOR with cellular hyperfunctions, then such a dual therapeutic approach may yield synergistic benefits.

## EXPERIMENTAL PROCEDURES

All the procedures involving animals were carried out in accordance with NIH guidelines and approved by either the University of Virginia or Jackson Labs West Institutional Animal Care and Use Committee.

### rhTFAM treatment of aged mice

Recombinant human Mitochondrial Transcription Factor A (rhTFAM) produced and purified by Gencia Corporation, (Charlottesville, VA) stored in 50% sorbitol, and dialyzed against 5% glycerol in 1x PBS then filtered through 10,000 MWCO centrifugal filters just before injection. Twenty month-old male C57Bl/6 mice procured from Jackson Laboratory were injected intravenously (tail vein) with rhTFAM. Electrophoretic mobility shift assay (EMSA) was done to measure the DNA binding capacity of the rhTFAM, and .67mg/kg was injected into each mouse (n=9) once per week for 5 weeks. Equal numbers (n=9) of mice were injected with buffer control in a paired design.

### Rotarod Training and Running Wheels

Mice were pre-trained on 4-lane rotarod (Columbus Instruments) with 3.0 cm. diameter rod. They were placed in pairs (injected with rhTFAM or buffer CTL) on the rod that smoothly accelerated from 0 to 40 rpm with the increments of 1 rpm increase every 5 seconds. Three training sessions were conducted with 15 minutes rest period between runs, with food and water provided. The mice were placed individually in standard mouse cages fitted with running wheels (Wheel Counter; Columbus Instruments) for 12 hours (6.00 PM to 6.00 AM). Mice were acclimatized 1 week before the experiment and normal food and water was provided.

### Mitochondrial respiration

One week after the 5th injection of rhTFAM the mice were euthanized in pairs (rhTFAM and buffer CTL) by CO2 inhalation, and their brain, heart and about 1gm of skeletal muscle (quadriceps) were removed and homogenized in mannitol-sucrose buffer [21]. About 1 ml was saved in −80C for protein and nucleic acid extraction. Crude mitochondria were isolated by differential centrifugation method and the P2 pellets were dissolved in 2.4 ml MirO5 (http://www.oroboros.at/index.php?id=524#857) buffer and 0.6 ml of this was used for measuring the respiration in Oxygraph. The same tissue samples from buffer CTL and rhTFAM treatments were run side by side with sequential addition of substrates to assay complex I through complex IV respiration as described elsewhere [21].

### Nucleic acid extraction and RT-qPCR

Total genomic DNA and RNA were extracted from the total tissue homogenates of brain, heart and skeletal muscle using All-Prep Kits from Qiagen and were quantified by NanoDrop 2000C spectrophotometer. One μg RNA was reverse transcribed to cDNA using iScript, (BioRad) and random hexamer primers. Primers and TaqMan probes used for multiplex qPCR assays, or primer pairs for SybrGreen assays, were designed using Beacon Designer software, and the qPCR assays were performed on a CFX 96 Real Time System (C1000 Thermal Cycler, BioRad). Mouse brain cDNA was generated and used as external standards for the nuclear genes assayed. Mouse mtDNA circles were prepared from mouse liver gDNA by treating with Plasmid-Safe ATP-Dependent DNase (Epicentre Biotechnologies) and were used as standards to quantify the mitochondrial genes. Mouse liver gDNA was used as external standard to assay some of the common multiple house keeping genes like GAPDH, beta Actin and 18s rRNA in the samples and their geometrical mean was used to normalize the gene copy numbers and gene expression levels of the genes we assayed.

In a separate, previously reported, experiment young adult (4-5 month old) male C57BL/6 mice (n=6) were injected IV through tail veins with dialyzed rhTFAM capable of binding 100ug of DNA (~450 ug) and equal numbers were given control buffer [21, 24]. These mice had been exposed to weekly rotarod testing using the same rotarod instrument but a more intense training regimen [21, 24]. The total gDNA and RNA were similarly extracted from their brain, heart and skeletal muscle and qPCR assays were done using the gDNA and cDNA generated from the RNA. These gDNA and cDNA samples were used in the present study.

Real time PCR assays were done with gDNA and cDNA from young and old mice treated with control buffer or the young and old mice treated with rhTFAM to see if there is any difference in mitochondrial gene copy numbers or in mitochondrial biogenesis because of aging. We assayed 16s rRNA, CO1, CO3 and CytB for mitochondrial DNA and to know the impact of aging on mitochondrial biogenesis PGC-1α, NRF1, TFAM and TFMB2 levels were determined. The levels POLg, POLRMT and SirT3 levels were also assayed for the brain cDNA.

### Morris Water Maze in Aged Mice

In a separate study, forty-five, 20 month old C57BL/6J mice were divided into three groups, vehicle, low and high Dose rhTFAM (.3 and 1 mg/kg, respectively). The mice were grouped by mean body weight per cage, and were dosed weekly via IV for a total of 4 doses. Dosing did not exceed 1% v/bw. Body weights and clinical observations were recorded weekly at the time of dosing. During Week 5, Morris water maze (MWM) training was initiated. Water temperature was 22°C. Mice that sank or floated were removed from the study. Mice were habituated to the water maze by placing each subject into the apparatus for 60 seconds with no opportunity to escape. Commencing on the following day, the mice were trained in the hidden platform version of the maze. The platform was submerged below the surface of the water, and the mouse was placed in the water and given 60 seconds to locate the platform. Once on the platform, mice were allowed to remain for 15 seconds before being returned to their cage. For mice that did not locate the platform in 60 seconds, they were guided to the platform and allowed to remain on the platform for 15 seconds before being returned to their cage. Mice were padded dry between MWM trials and before being returned to the cage. Each mouse underwent four successive trials a day, with 10 minute intervals, for 7 days. The sequence of water entering points differed for each of the 4 trials each day, but the location of the platform remained constant throughout the study. Latency (seconds) was averaged by each run (4 runs per timepoint), and then by treatment group.

To measure the strength of spatial memory retention, on the 8th day, a ‘probe test’ was conducted. Mice were placed in the water to swim freely for 60 seconds without the platform and the time spent in the quadrant that had contained the platform, and the number of crosses over the previous position of the platform, was recorded. The probe trial was repeated 15 days later.

### Morris Water Maze in Young Mice

In a separate study, twenty (20) 8-10 week old C57BL/6J mice were divided into two groups, Vehicle and rhTFAM (.3 mg/kg). The mice were grouped by mean body weight per cage, and were dosed weekly via IV for a total of 4 doses. Dosing did not exceed 1% v/bw. Body weights and clinical observations were recorded weekly at the time of dosing. During Week 5, Morris water maze testing was initiated. Water temperature was 22°C. Mice that sank or floated were removed from the study. Mice were habituated to the water maze by placing each subject into the apparatus for 60 seconds with no opportunity to escape. Commencing on the following day, the mice were trained in the hidden platform version of the maze. The platform was submerged below the surface of the water, and the mouse was placed in the water and given 60 seconds to locate the platform. Once on the platform, mice were allowed to remain for 15 seconds before being returned to their cage. For mice that did not locate the platform in 60 seconds, they were guided to the platform and allowed to remain on the platform for 15 seconds before being returned to their cage. Mice were padded dry between MWM trials and before being returned to the cage. Each mouse underwent four successive trials a day, with 10 minute intervals, for 7 days. The sequence of water entering points differed for each of the 4 trials each day, but the location of the platform remained constant throughout the study. Latency (seconds) was averaged by each run (4 runs per timepoint), and then by treatment group.

### Western blotting

Twenty-four hours following the final probe trial, the mice were sacrificed. Brains were lysed and P2 mitochondria-enriched fractions prepared. We determined the effects of rhTFAM on individual brain electron transport chain subunits. Four micrograms of brain P2 fractions from each brain were run on SDS-PAGE and transferred for blotting. Using the MitoProfile® Total OXPHOS rodent antibody cocktail (ab110413, Abcam), optical density units of the five bands were totaled.

In other experiments, whole brain protein was isolated, electrophoresed and probed with antibodies to BDNF, PGC-1 alpha, synapsin or PSD-95. Results were normalized to beta actin levels.

### Complex I and IV Activities

The aged mouse brain P2 mitochondria enriched fractions were assayed for Complex I and IV activities. To determine the activity of mitochondrial complex I, the microplate assay kit for complex I activity (MitoSciences, MS141) was used. Mitochondrial proteins (5.5 mg/mL) were extracted by adding 1/10 volume of lauryl maltoside, and 50 μg of proteins were used for the assay. Complex I was immunocaptured on microplates, and the activity was determined by the oxidation of NADH to NAD+. Complex I activity was measured by the increase in absorbance at 450 nm and expressed as the change in absorbance per minute per microgram of protein.

Mitochondrial complex IV activity was determined with the microplate assay kit for complex IV activity (MitoSciences, MS444). Mitochondrial proteins (5.5 mg/mL) were extracted by adding 1/10 volume of lauryl maltoside, and 75 μg of proteins were used for the assay. Complex IV was immunocaptured on microplate, and activity was determined by following the oxidation of reduced cytochrome C as revealed by a change in absorbance at 550 nm. Complex IV activity was expressed as the change in absorbance per minute per microgram of protein.

### ATP assay

The Cell Titer-Glo® Luminescent Cell Viability Assay kit was used for ATP assay following the manufacturer's instruction. Briefly, the assay buffer and substrate were equilibrated to room temperature, and the buffer was transferred to and gently mixed with the substrate to obtain a homogeneous solution. After a 30 min equilibration of the plate to room temperature, 100 μl of the assay reagent was added into each well containing brain P2 mitochondrial pellets and the content was mixed for 2 min. After 10 min incubation at room temperature, the luminescence was read on a PHERAstar FS Reader (BMG Labtech Cary, NC). The substrate mix contains luciferase, which in the presence of Mg2+ and ATP converts luciferin into oxiluciferin and concomitantly releases energy in the form of luminescence. Signal strength is directly proportional to the amount of ATP present.

### TBARS assay

Brain lysates were also studied for levels of TBARS (Thiobarbituric Acid Reactive Substances) a measure of ROS driven lipid peroxidation using the OxiSelect TBARS assay kit (Cell Biolabs).

### Statistical analyses

We used SigmaPlot, SigmaStat, GraphPad InStat and Prism softwares for statistical analyses. Both parametric and non-parametric tests were used, depending on whether data distributed normally. Matched-pair t-tests were used where appropriate.
